# A Post-Graduate College for London

**Published:** 1898-05-28

**Authors:** 


### A Post-Graduate College for London.

At last there seems to be a great probability of our seeing established in London a centre for post-graduate teaching—a post-graduate medical college in fact, where men who have just passed their examinations may continue their medical education, and put themselves in touch with a wider circle of medical thought than was possible while they were attached to a single hospital, and where those who have already been in practice may come for that fresh study and that renewal of knowledge which is every year becoming more and more necessary if men are to keep themselves abreast of the medicine of the day, and if they are to hold their own against the crowd of younger competitors which springs up so continuously around them. It is, we understand, proposed to found an institution to be styled the Medical Graduates College and Hospital. The College will consist of: (1) A building containing lecture theatres, class-rooms, laboratories, clinical museum, library, reading-room, &c. (2) A clinical hospital containing a limited number of beds, with an out-patient clinic. It is not contemplated that the patients shall be admitted into the hospital for prolonged treatment, and it is intended that medical men of eminence connected with all the existing schools shall conduct public consultations, and shall undertake the clinical teaching. Medical men having patients amongst the poorer classes, for whom they desire further advice, will be invited to bring them up for consultation at these clinics, and the staff will deliver a diagnosis and a scheme of treatment, and will inform the practitioner where it can be best carried out.

It is quite clear at the outset that such a college, if properly worked, would tend to fill what at present is a great hiatus in the medical institutions of the metropolis, and that it would make London a centre of attraction to medical men from all parts of the world, who now wander off to other places where they can get what London with its enormous wealth of clinical material has hitherto failed to provide for them. It is equally clear that if badly managed, or if managed with selfish aims, it may become the source of much evil and the centre of much that is objectionable. We are glad, therefore, to hear that the scheme has already received the approval of some who occupy the highest positions in the Royal Colleges, and promises of substantial guarantees from many others, and we would strongly urge that the leaders of the profession should go further, and should take an active, perhaps a controlling part in its management. Under their sanction, and with their guarantee of professional propriety, there seems no reason why such a college should not attract the cream of the younger medical teachers, and for such ample scope will be provided. But the best of them will not stir a hair's breadth unless assured that their course is professionally correct, while if the affair got into the hands of any but the best it might lead to regrettable consequences. We would therefore urge upon our leaders the great desirability of their taking up the matter from the beginning. The project is the outcome of a public demand, which has already been met in other parts of the world. The Post-Graduate College, in some form or another, is bound to come sooner or later. Let it then come in a form which shall redound to the honour of the profession.

